# Lipoyltransferase 1 Gene Defect Resulting in Fatal Lactic Acidosis in Two Siblings

**DOI:** 10.1155/2016/6520148

**Published:** 2016-05-10

**Authors:** Véronique Taché, Liga Bivina, Sophie White, Jeffrey Gregg, Joshua Deignan, Simeon A. Boyadjievd, Francis R. Poulain

**Affiliations:** ^1^Department of Obstetrics and Gynecology, Division of Maternal Fetal Medicine, University of California Davis, Sacramento, CA 95817, USA; ^2^Department of Pediatrics, Division of Genomic Medicine, University of California Davis, Sacramento, CA 95817, USA; ^3^Department of Pediatrics, Division of Neonatology, University of California Davis, Sacramento, CA 95817, USA; ^4^Department of Pathology and Laboratory Medicine, University of California Davis, Sacramento, CA 95817, USA; ^5^Department of Pathology and Laboratory Medicine, University of California Los Angeles, Los Angeles, CA 90024, USA

## Abstract

A term male neonate developed severe intractable lactic acidosis on day of life 1 and died the same day at our institution. The family previously lost another term, female newborn on day of life 1 from suspected sepsis at an outside hospital. After performing an autopsy on the neonate who died at our institution, extensive and lengthy neonatal and parental genetic testing, as well as biochemical analyses, and whole exome sequencing analysis identified compound heterozygous mutations in the lipoyltransferase 1 (*LIPT1)* gene responsible for the lipoylation of the 2-keto dehydrogenase complexes in the proband. These mutations were also identified in the deceased sibling. The clinical manifestations of these two siblings are consistent with those recently described in two unrelated families with lactic acidosis due to* LIPT1* mutations, an underrecognized and underreported cause of neonatal death.* Conclusions*. Our observations contribute to the delineation of a new autosomal recessive metabolic disorder, leading to neonatal death. Our case report also highlights the importance of an interdisciplinary team in solving challenging cases.

## 1. Introduction

Neonatal deaths (4.05 per 1,000 US births in 2010) [1] are devastating for families and the providers. The leading causes of deaths are congenital malformations and conditions related to prematurity, but often no definitive etiology can be determined.

Our case report details the investigation of two consecutive infants, born to a nonconsanguineous couple after full-term, otherwise uncomplicated pregnancies, who died on their first day of life. These two neonatal deaths were ultimately linked to mutations in the lipoyltransferase 1 (*LIPT1)* gene. Lipoyltransferase 1 is one of several known proteins involved in the biosynthesis and function of lipoic acid. Lipoic acid is an essential cofactor to the activity of mitochondrial enzymes that comprise four 2-oxoacid dehydrogenases and the glycine cleavage system [2].

## 2. Case Presentation

### 2.1. Case  1

A 2.75 kg term female infant was delivered via uncomplicated vaginal delivery to a healthy 24-year-old G1 Mexican-American woman. The only pregnancy complication was maternal hypothyroidism. The infant had Apgar scores of 5, 6, and 6 at 1, 5, and 10 minutes, respectively. Cord gases showed moderate metabolic acidosis (venous: pH of 7.16, PCO_2_ of 60, PO_2_ of 8.6; arterial blood gas: pH 7.14, PCO_2_ 60.4, and PO_2_ 9). The infant's condition rapidly deteriorated, with progressive respiratory distress and shock. The infant ultimately died at 1.5 hours of life. Maternal group B streptococcus screen, resulting after the neonatal death, was negative. An autopsy detected no abnormality and the placental gram stain was negative. The cause of death ascribed by the coroner's office was chorionitis due to maternal group B streptococcus.

### 2.2. Case  2 (Proband)

In light of her prior pregnancy outcome, the mother was referred to our high-risk obstetrical clinic for her second pregnancy. Universal carrier screening showed her to be a carrier for glycogen storage disease type V and congenital disorder of glycosylation type 1a. The father's carrier screening for these two conditions was negative. The pregnancy was unremarkable other than maternal hypothyroidism, which was well controlled.

The patient was born vaginally at our hospital, after induction of labor for nonreassuring fetal heart tracing and decreased fetal movements. The 3.5 kg infant was vigorous at birth and Apgar scores were 8 and 8 at 1 and 5 minutes, respectively. Initial physical examination was unremarkable. Cord gases were not obtained.

Within two hours of life, the infant developed progressive respiratory distress. Examination revealed gasping respirations and poor perfusion. Capillary blood gas showed severe metabolic acidosis (pH of 6.80, PCO_2_ of 65, and BE of −27). Chemistry showed blood sugar of 56 mg/dL, Na^+^ 145 meq/L, K^+^ 3.9 meq/L, Cl^−^ 116 meq/L, CO_2_ < 5 meq/L, BUN 6 mg/dL creatinine 1.4 mg/dL, lactate 16 mmol/L, pyruvate 0.346 mmol/L, ammonia 183 *μ*mol/L, AST 141 U/L, and ALT 15 U/L. Complete blood count, chest X-ray, and electrocardiogram were unrevealing. Sepsis evaluation ultimately proved negative.

Immediate management consisted of tracheal intubation, umbilical vessels catheterization, administration of broad-spectrum antibiotics, fluids, a cardiopressor, and alkali with vitamin B1, with B12 and levocarnitine therapy empirically started. Alprostadil infusion was discontinued after echocardiography showed normal heart and great vessel anatomy. Hospital course was one of unremitting shocks that failed to respond to therapeutic measures. Lactate concentration was 29 mmol/L at 15 hours of age. The infant expired at 18 hours of age, shortly after being placed on hemodialysis.

Gross and pathological autopsy examination did not reveal any diagnostic abnormalities. Newborn screen results, collected before 12 hours of age, showed tandem mass spectroscopy abnormalities in the acylcarnitine panel, suggestive of CPT-1 deficiency. Additionally, laboratory tests were performed to determine possible etiologies (Table 1). Parents consented to whole exome sequencing (WES), using DNA samples from patient and both parents, approximately 2 years after the death of the proband. Results of WES showed that the deceased infant was compound heterozygous for two nonsense mutations in the* LIPT1* gene (c.806G>A and c.980T>G). Mutations were in trans, as the c.806G>A was paternally inherited and the c.980T>G mutation was maternally inherited.

To ascertain whether the* LIPT1* gene mutations were present in the previously deceased child, we obtained formalin fixed paraffin embedded autopsy tissue from the liver and kidney from the coroner's office, extracted DNA from the blocks, and performed targeted* LIPT1* mutation analysis. The same* LIPT1* gene mutations were found in Case  1, leading us to believe these gene variants were the cause of the neonates' death.

### 2.3. Case  3

While the genetic evaluation of her deceased children was ongoing, the mother became pregnant while on birth control. We were able to perform chorionic villous sampling with targeted testing of the identified* LIPT1* mutations. The results showed the fetus did not carry any* LIPT1* mutation previously detected in the siblings. The third pregnancy was again only complicated by maternal hypothyroidism, which was well controlled. She delivered a full-term 4.2 kg female infant, who is alive and thriving at 12 months of life. Interestingly, the mother reported significantly more fetal activity with this pregnancy than in her prior 2 pregnancies.

## 3. Discussion

Most common etiologies of neonatal shock include hypovolemia, asphyxia, cardiac defects, and sepsis. Although less frequent, inborn errors of metabolism can lead to severe end-organ dysfunction and neonatal shock [3, 4].

Lactic acidosis is conventionally defined as the concurrence of lactate accumulation in excess of 5 mmol/L and a pH < 7.35. While serum lactate can be elevated in a number of inborn errors of metabolism such as glycogen storage diseases and fatty acid oxidation disorders, metabolic disorders associated with severe lactic acidosis point to abnormalities of pyruvate metabolism or respiratory chain function [4, 5]. Whether it is the result of inadequate tissue oxygenation or due to inborn metabolic disorders, severe and prolonged lactic acidosis and the resulting multiorgan energy failure may prove fatal.

Lipoic acid is an essential cofactor to several cellular redox reactions and is found in all kingdoms of life. In humans, lipoic acid is exclusively found in mitochondria and is essential to the activity of five different enzymatic complexes: pyruvate dehydrogenase, alpha ketoglutarate dehydrogenase, 2-oxoadipate dehydrogenase, branched chain ketoacid dehydrogenase, and the glycine cleavage system [2, 6, 7]. A number of proteins are necessary to the biosynthesis of lipoic acid and mutations in several genes are known to cause human diseases [2].

The lipoyl transferase, encoded by* LIPT1,* participates in the final steps of lipoate synthesis from octanoate and is essential to the activity of all aforementioned enzymatic complexes but the glycine cleavage system.

Tort et al. reported the first patient with mutations of the* LIPT1* gene [8]. After a two-day symptom-free period, the neonate presented with bradycardia and weight loss, and her clinical condition worsened over the next several days to include generalized hypertonia and dystonia and progressive organ failure leading to her death at 9 days of life. The sequence analysis of the* LIPT1* gene identified two heterozygous missense mutations (c.212C>T and c.292C>G). Shortly thereafter, Soreze et al. reported another case of* LIPT1* gene mutations in a male infant who developed Leigh disease after a gastroenteritis episode at 18 months of life [9]. This infant's early development was notable for delayed psychomotor development with hypotonia. Ultimately, the sequence analysis of* LIPT1* gene demonstrated compound heterozygous mutations (c.875C > G and c.535A > G) in* LIPT1* gene. Both patients had elevated urine lactate and ketoglutarate but normal serum glycine levels. We herein report a family with two successively affected neonates with mutations in* LIPT1* gene who died of intractable cardiovascular collapse on the first day of life. A third child, who did not have the* LIPT1* mutations that affected her siblings, is alive and well. The proband had profound lactic acidemia, elevated urine lactate, ketoglutarate, 2-oxoacids, and normal serum glycine. The biochemical profile of the proband is consistent with that observed in the two previously published cases of* LIPT1* mutations. The lack of glycine elevation suggests sparing of the glycine cleavage system, consistent with the fact that the glycine cleavage system does not depend on* LIPT1* for lipoylation [2].

The reason for heterogeneity of clinical presentation in these three reports is at present unclear. Mutations reported differ in all three cases. The infant reported by Tort et al. had compound heterozygous mutations resulting in substitution of Ser71 for Phe and of Arg98 for Gly, respectively [8]. The case reported by Soreze et al. had compound heterozygous mutations resulting in a stop codon and in the substitution of Thr179 for Ala [9]. The mutations seen in the present family introduced two stop codons. Residual activity of the lipoyltransferase enzyme, heterogeneity of the 2-keto dehydrogenase complexes activity in different tissues, and yet to be identified modifiers of enzyme activity may account for some of the varying clinical presentations. However, the clinical spectrum of patients with disorders of mitochondrial function is known to be broad and variable (any tissue, any symptom, and any age), as is illustrated in the results of recent Pediatric series [10–12]. As more cases are reported and the role of lipoic acid in mitochondrial metabolism is further delineated, we may be able to better understand the clinical spectrum of* LIPT1* gene mutations.

In summary, lipoyltransferase 1 gene defect is associated with severe mitochondrial dysfunction disrupting lipoic acid biogenesis. Clinical manifestations associated with defective lipoic acid metabolism include early seizures, hypotonia, cardiomyopathy and pulmonary hypertension, and Leigh-like encephalopathy. In the most extreme cases, they may result in cardiorespiratory collapse and early neonatal death.

## Figures and Tables

**Table 1:** Laboratory evaluation of the proband.

Testing	Test	Results
Genetic tests	Chromosomal microarray analysis	High resolution identified a copy number gain of chromosome band 14q24.3 spanning approximately 0.255 Mb in a nondisease associated region. This gain contains at least five genes and is expected to be inherited 93% of the time. Parental studies were not recommended.
	*CPT1A* gene sequencing	No known deleterious mutations
	Comprehensive mitochondrial DNA analysis	No large deletions or deleterious mutations
	*PC* gene sequencing (pyruvate carboxylase deficiency)	No known deleterious mutations
	*DLAT* gene sequencing (pyruvate dehydrogenase E2 deficiency)	No known deleterious mutations

Biochemical analyses	Mitochondrial respiratory chain enzyme analysis, skin fibroblasts	Deficiencies in mitochondrial electron transport chain enzymes not detected
	Newborn screen	Pattern of elevation consistent with *CPT1*, otherwise normal
	Plasma amino acids	Suggestive of lactic acidemia, very elevated alanine, and serum glycine was normal
	Pyruvic acid	Elevated
	Urine organic acids	Notable for lactic aciduria, elevated 2-OH and 2-oxoacids

*CPT1A/CPT1*: carnitine palmitoyltransferase 1A.

*PC*: pyruvate carboxylase.

*DLAT*: dihydrolipoamide S-acetyltransferase.

## Abbreviations

CDG-1a: Congenital disorder of glycosylation type 1a
GBS: Group beta streptococcal
GSD-V: Glycogen storage disease type V
*LIPT1* gene: Lipoyltransferase 1 gene
WES: Whole exome sequencing.
